# Children’s rates of COVID-19 vaccination as reported by parents, vaccine hesitancy, and determinants of COVID-19 vaccine uptake among children: a multi-country study from the Eastern Mediterranean Region

**DOI:** 10.1186/s12889-022-13798-2

**Published:** 2022-07-18

**Authors:** Moawiah Khatatbeh, Samir Albalas, Haitham Khatatbeh, Waleed Momani, Omar Melhem, Omar Al Omari, Zeinab Tarhini, Ashraf A’aqoulah, Mohammed Al-Jubouri, Abdulqadir J. Nashwan, Ghaleb Adwan, Zaid Altaany, Ayat Nashwan, Khaled Al-Waqfi, Lujain Abuirsheid, Raghad Ayasreh, Mohammed Al Mutairi, Ala’a B. Al-Tammemi

**Affiliations:** 1 Department of Basic Medical Sciences, Faculty of Medicine, Yarmouk University, Irbid, Jordan; 2 Department of Health Services Administration, Yarmouk University, Irbid, Jordan; 3 Doctoral School of Health Sciences, Faculty of Health Sciences, University of Pécs, Pécs, Hungary; 4 Department of Nursing, Fatima College of Health Sciences, Abu Dhabi, United Arab Emirates; 5 College of Nursing, Sultan Qaboos University, Muscat, Oman; 6 CAPTuR Laboratory, Control of Cell Activation in Tumor Progression and Therapeutic Resistance, Limoges, France; 7 Institute of Epidemiology and Tropical Neurology, GEIST, Limoges, France; 8 Department of Health Systems Management, College of Public Health and Health Informatics, King Saud Bin Abdulaziz University for Health Sciences, Riyadh, Saudi Arabia; 9 King Abdullah International Medical Research Center, Riyadh, Saudi Arabia; 10 College of Nursing, University of Baghdad, Baghdad, Iraq; 11 Nursing Department, Hazm Mebaireek General Hospital, Hamad Medical Corporation, Doha, Qatar; 12 Department of Biology and Biotechnology, An-Najah National University, Nablus, Palestine; 13 Department of Sociology and Social Work, Yarmouk University, Irbid, Jordan; 14 Ministry of Health, Kuwait, Kuwait; 15 Migration Health Division, International Organization for Migration (IOM), Amman, Jordan

**Keywords:** Arab countries, attitude, COVID-19, hesitancy, coverage, vaccination, EMR

## Abstract

**Background:**

Huge efforts are being made to control the spread and impacts of the coronavirus pandemic using vaccines. However, willingness to be vaccinated depends on factors beyond the availability of vaccines. The aim of this study was three-folded: to assess children’s rates of COVID-19 Vaccination as reported by parents, to explore parents’ attitudes towards children’s COVID-19 vaccination, and to examine the factors associated with parents’ hesitancy towards children’s vaccination in several countries in the Eastern Mediterranean Region (EMR).

**Methods:**

This study utilized a cross-sectional descriptive design. A sample of 3744 parents from eight countries, namely, Iraq, Jordan, Kuwait, Lebanon, Palestine, Qatar, Saudi Arabia (KSA), and the United Arab Emirates (UAE), was conveniently approached and surveyed using Google forms from November to December 2021. The participants have responded to a 42-item questionnaire pertaining to socio-demographics, children vaccination status, knowledge about COVID-19 vaccines, and attitudes towards vaccinating children and the vaccine itself. The Statistical Package for Social Sciences (SPSS- IBM, Chicago, IL, USA) was used to analyze the data. A cross-tabulation analysis using the chi-square test was employed to assess significant differences between categorical variables and a backward Wald stepwise binary logistic regression analysis was performed to assess the independent effect of each factor after controlling for potential confounders.

**Results:**

The prevalence of vaccinated children against COVID-19 was 32% as reported by the parents. Concerning parents’ attitudes towards vaccines safety, about one third of participants (32.5%) believe that all vaccines are not safe. In the regression analysis, children’s vaccination was significantly correlated with parents’ age, education, occupation, parents’ previous COVID-19 infection, and their vaccination status. Participants aged ≥50 years and those aged 40-50 years had an odds ratio of 17.9 (OR = 17.9, CI: 11.16-28.97) and 13.2 (OR = 13.2, CI: 8.42-20.88); respectively, for vaccinating their children compared to those aged 18-29 years. Parents who had COVID-19 vaccine were about five folds more likely to vaccinate their children compared with parents who did not receive the vaccine (OR = 4.9, CI: 3.12-7.70). The prevalence of children’s vaccination in the participating Arab countries is still not promising.

**Conclusion:**

To encourage parents, vaccinate their children against COVID-19, Arab governments should strategize accordingly. Reassurance of the efficacy and effectiveness of the vaccine should target the general population using educational campaigns, social media, and official TV and radio channels.

**Supplementary Information:**

The online version contains supplementary material available at 10.1186/s12889-022-13798-2.

## Background

The Coronavirus Disease-2019 (COVID-19) was announced as a Public Health Emergency of International Concern (PHEIC) in January 2020, with 5.5 million deaths reported until January 2022 [1]. Most countries, around the World, have witnessed large numbers of cases despite the precautions taken [2]. For instance, the total number of the reported COVID-19 cases has exceeded 10% of the total population in 115 countries [3]. This, in turn, have led to severe impact including psychological burden [4, 5]. Efficacious vaccine is an essential tool in the fight against the current COVID-19 pandemic to achieve collective immunity, which can help reduce transmission, hospitalizations, and intensive care utilization, as well as prevent additional mortality [6]. An up-to-date Italian study found that COVID-19 vaccines can prevent approximately 17% of expected cases, 32% of hospitalizations, 29% of admissions to intensive care units, and 38% of deaths [7].

Several COVID-19 vaccines are now available for public. However, major issues have been identified to affect vaccine coverage, including limited manufacturing capacity, global inequality in the distribution of COVID-19 vaccines, and most importantly vaccine hesitancy which could represent a major challenge in the global efforts to control the pandemic [8, 9]. Vaccine hesitancy refers to a delay in accepting or refusing vaccination despite its availability [10].

Many studies around the world have reported hesitancy towards COVID-19 vaccine among the public and this was reflected in a systematic review involved 31 studies published in 2020 [11]. One-third of participants in a study from four African and Middle Eastern countries were hesitant about getting vaccinated against the COVID-19 [12]. Similarly, another multi-country study showed that around one-third of respondents from Bangladesh and India, and around one-quadrant of respondents from Pakistan and Nepal are hesitant about getting vaccinated against the COVID-19 [13]. Likewise, a Japanese study showed that approximately one-third of respondents are hesitant about getting vaccinated against the COVID-19 [14]. Arab countries also reported similar trends in COVID-19 hesitancy [15–17]. Certainly, individuals who are hesitant in getting vaccinated query the usefulness and safety of vaccines for their children [18]. In Italy, a cross-sectional study was conducted to evaluate the knowledge, attitude, and intention to vaccinate children < 18 years. They found that 41.2% of families of children ≥12 years and 36.1% among families of children < 12 years would not vaccinate their children [18]. Perceived vaccine safety and efficacy and perceived risk of transmission of infection to adults were the two determinants of intention to vaccinate for both age groups [18].

Many factors influence vaccination decisions, including trust in the government and health care professionals, social influences, high levels of knowledge about the vaccine, and general positive attitudes toward vaccines [19]. Another study found that parents with different levels of education and employment status had significant differences in vaccine knowledge and awareness; similarly, these two factors also significantly influenced parental vaccine hesitancy [20]. The knowledge score of parents was inversely correlated with vaccine hesitancy and moderately associated with their awareness score [20].

In a study conducted in Poland regarding children vaccination, the main concerns expressed by parents were the possibility of adverse effects, the lack of adequate testing of the preparations in children, and the fear of future complications [21]. Therefore, the COVID-19 vaccine must have proven safety and efficacy in preventing complications as well as transmission to ensure vaccination of children [22].

In the Arab countries, like Jordan, commitment to precaution measures against COVID-19 virus such as hand washing, wearing a face mask, and social distancing is not optimal [23]. Figures of the vaccinated population against COVID-19 in Arab countries, including children, are still not promising. To our knowledge, no studies have been conducted in these countries to assess the attitudes of parents towards children vaccination against COVID-19 infection. At the same time, official data on the rates of vaccinated children in the participating countries are lacking despite its availability since Arab countries were first to approve Chinese COVID-19 vaccine [24]. The aims of this study were to (1) assess the parents’ self-reported coverage of children’s vaccination against COVID-19, (2) assess parents’ attitudes toward children’s vaccination, and (3) study factors associated with vaccination’s status and hesitancy of parents towards children’s vaccination in several Arab countries. It is important to stress the point that all the countries involved in our study had almost similar response to the COVID-19 pandemic, including lockdowns, quarantine, as well as non-pharmaceutical interventions including physical distancing, public face masking, hand hygiene, and practicing respiratory etiquette. Additionally, these countries made huge efforts to make COVID-19 vaccines available in their respective communities.

## Methods and materials

### Study setting, design, and participants

Our study was conducted in eight countries located in the Eastern Mediterranean Region (EMR), namely: Iraq, Jordan, Kuwait, Lebanon, Palestine, Qatar, Saudi Arabia (KSA), and the United Arab Emirates (UAE). A descriptive questionnaire-based cross-sectional online survey was employed in this study focusing on married individuals having children (parents). A convenience sample was approached through social media platforms. A questionnaire was firstly developed in English, then it was translated into Arabic (the native language of our respondents) by two bilingual specialists. The questionnaire was then uploaded to Google Form® and disseminated to those who could access the online survey. The inclusion criteria included being a citizen of any participating country, aged 18 or older, married and having children, reading, and understanding Arabic, and being willing to fill the online questionnaire. The google form led participants who were single or not having children to submit their responses at early stage and their responses were excluded. An online link of the questionnaire, along with an introductory letter about the study, was sent to participants via media platforms like Facebook®, WhatsApp®, Twitter®, and email addresses, for a period of four weeks; 15-November- 13-December 2021. In addition, respondents were asked to share the questionnaire link with their relatives, friends, and social networks.

### Study instrument

The authors reviewed the available literature and developed a 42-item questionnaire composed of three main sections (Supplementary File). The first section consisted of 12 questions on participants’ socio-demographic characteristics such as age, gender, marital status, having children, education, nature of work or study, and country of residence. The second section comprised four questions that solicited general knowledge about the COVID-19 vaccine. The third section of the questionnaire consisted of 26 items and inquired about participants’ attitudes towards COVID-19 vaccination of their children and the vaccine itself.

### Survey development

Two researchers checked the content of the questionnaire and its face validity before the final approval. To ensure its reliability, the questionnaire was pilot tested with the first 40 responses. Based on these responses and the feedback, refinements were made. The Cronbach’s alpha score was found to be 0.81. The responses of the pilot-testing were excluded from the final analysis.

### Ethical considerations

All participants have given their informed consent through reading the following statement and ticking a box next to it: “Completing the questionnaire would be considered consent to voluntary participation”. Participants were informed that the study would not disclose any personal information and that their data would be stored under high-security settings with only the research team having access to these data.

### Statistical analysis

The Statistical Package for Social Sciences (SPSS- IBM, Chicago, IL, USA) was used to analyze the data. Categorical variables were reported as frequency counts and percentages. A cross-tabulation analysis using the chi-square test was employed to assess significant differences between categorical variables. Finally, all statistically significant factors revealed from the cross-tabulation analysis were subjected to a backward Wald stepwise binary logistic regression analysis to assess the independent effect of each factor after controlling for potential confounders. A *P* value < 0.05 was set for statistical significance.

## Results

### Participants’ characteristics

A total of 3744 participants with valid responses were involved in the final analysis. More than half of the sample (55.5%) was females (*n* = 2078) and 44.5% (*n* = 1666) was males. About 40% of the study population have been diagnosed with COVID-19 infection and the majority (83%, *n* = 3108) have been vaccinated against the infection. The socio-demographic characteristics of the study population are shown in Table 1.

**Table 1:** Socio-Demographic Characteristics of the Study Participants (*N* = 3744)

Characteristic	n (%)
**Gender**	
Male	1666 (44.5)
Female	2078 (55.5)
**Age (years)**	
18–29	331 (8.8)
30–39	1561 (41.7)
40–49	1340 (35.8)
> 50	512 (13.7)
**Work or study field**	
None health-related	2679 (71.6)
Health-related	1065 (28.4)
**Education**	
Secondary or less	544 (14.5)
≥ Graduate	3200 (85.5)
**Received a COVID-19 vaccine**	
No	636 (17.0)
Yes	3108 (83.0)
**Country of Residence**	
Jordan	997 (26.6)
KSA	419 (11.2)
UAE	333 (8.9)
Kuwait	475 (12.7)
Lebanon	424 (11.3)
Palestine	422 (11.3)
Qatar	306 (8.2)
Iraq	368 (9.8)
**Previous Diagnosis of COVID-19**	
No	2223 (59.4)
Yes	1521 (40.6)

### Prevalence of children vaccination against COVID-19 and parents’ beliefs about COVID-19 vaccine safety

**Fig. 1:**
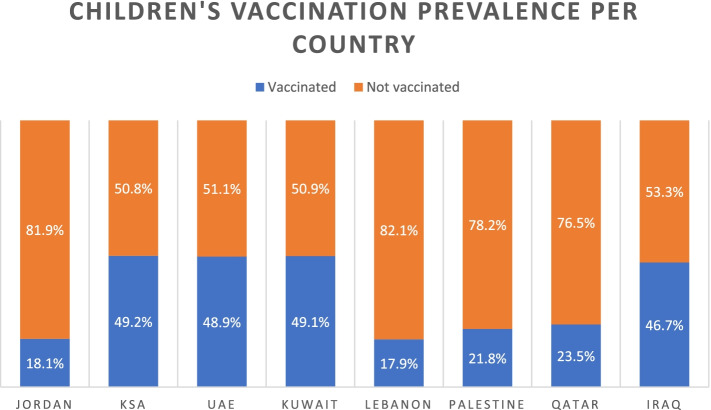
Prevalence of children vaccination against COVID-19 per country

Table 2 shows the number of children, their age, and beliefs of the study population towards the COVID-19 vaccine for children. About one-third of the sample (32.5%) reported that all COVID-19 vaccines are not safe and close percentage reported that their children received the vaccine (31.9%, *n* = 1194). Also, Fig. 1 shows children’s vaccination prevalence per country.

**Table 2:** Number of children, their age, and beliefs of parents towards COVID-19 vaccine for the children (*N* = 3744)

Characteristic	n (%)
**Number of children**	
1	578 (15.4)
2	1022 (27.3)
≥ 3	2144 (57.3)
**Age of children/year**	
All kids are younger than 12	1817 (48.5)
All kids between 12 and 17	579 (15.5)
I have kids in both above categories	1348 (36.0)
**If any of your kids had COVID-19 vaccine**	
No	2550 (68.1)
Yes	1194 (31.9)
**Will the freedom to choose the type of vaccine affect your decision to vaccinate your children?**	
No	1211 (32.3)
Yes	1361 (36.4)
Maybe	1172 (31.3)
**In your opinion, are COVID-19 vaccines safe?**	
All vaccines are safe	2527 (67.5)
All vaccines are not safe	1217 (32.5)

### The relationship between participants’ socio-demographic characteristics and vaccination status of their children

To assess the impact of socio-demographic characteristics on the vaccination against COVID-19 among children, a cross-tabulation analysis was performed. This analysis revealed that all participant’s socio-demographic factors were associated with vaccination status of their children. These factors included: participant’s gender (*p* = 0.037), participant’s education (*p* <  0.001), work or study field (*p* <  0.001), participant’s age (*p* <  0.001), number of children (*p* <  0.001), children age (*p* <  0.001), if the parent had a COVID-19 before (*p* <  0.001), and if the parent received a COVID-19 vaccine (*p* <  0.001).

As shown in Table 3, parents whose work or study was health-related showed a higher tendency for not vaccinating their children compared to others. Moreover, willingness to vaccinate increased with an increased parents’ age. Remarkably, less than 4% of parents whose children were younger than 12 years have vaccinated their children.

**Table 3:** Cross-tabulation of socio-demographic factors associated with COVID-19 vaccination among children (*N* = 3744)

Variable	Children vaccination status		***P*** value٭
	No (%)	Yes (%)	
**Gender**			**0.037**
Male	1105 (66.3)	561 (33.7)	
Female	1445 (69.5)	633 (30.5)	
**Participant’s education**			**< 0.001**
Secondary or less	311 (57.2)	233 (42.8)	
≥ Graduate	2239 (70.0)	961 (30.0)	
**Work or study field**			**< 0.001**
None health-related	1770 (66.1)	909 (33.9)	
Health-related	780 (73.2)	285 (26.8)	
**Participant’s age/year**			**< 0.001**
18-29	309 (93.4)	22 (6.6)	
30-39	1335 (85.5)	226 (14.5)	
40-40	690 (51.5)	650 (48.5)	
≥ 50	216 (42.2)	296 (57.8)	
**Number of children**			**< 0.001**
1	458 (79.2)	120 (20.8)	
2	837 (81.9)	185 (18.1)	
≥ 3	1255 (58.5)	889 (41.5)	
**Children age**			**< 0.001**
All kids are younger than 12	1747 (96.1)	70 (3.9)	
All kids are between 12 and 17	254 (43.9)	325 (56.1)	
I have kids in both above categories	549 (40.7)	799 (59.3)	
**Had COVID-19 before**			**< 0.001**
No	1462 (65.8)	761 (34.2)	
Yes	1088 (71.5)	433 (28.5)	
**Received a COVID-19 vaccine**			**< 0.001**
No	231 (90.2)	25 (9.8)	
Yes	2011 (64.7)	1097 (35.3)	
I intend to get the vaccine	134 (77.5)	39 (22.5)	
I do not intend to get the vaccine	174 (84.1)	33 (15.9)	

٭Chi square test

As seen in Table 3, parents who have been vaccinated against COVID-19 and those who intend to get the vaccine in the future were more likely to have vaccinated children. With respect to the country of residence and perceived necessity for COVID-19 vaccine with vaccination status among children, parents living in KSA, UAE, Kuwait, and Iraq were more willing to vaccinate their children compared to parents from Jordan, Lebanon, Palestine, and Qatar. Furthermore, parents who believed that the vaccine is necessary for individuals younger than 18 years were more willing to vaccinate their children than those who didn’t have the same belief. Table 4 illustrates these associations.

**Table 4:** Cross-tabulation of vaccine related factors and country of residence with COVID-19 vaccination among children (*n* = 3744)

Variable	Children vaccination status		***P*** value٭
	No (%)	Yes (%)	
**Country of residence**			**< 0.001**
Jordan	817 (81.9)	180 (18.1)	
KSA	213 (50.8)	206 (49.2)	
UAE	170 (51.1)	163 (48.9)	
Kuwait	242 (50.9)	233 (49.1)	
Lebanon	348 (82.1)	76 (17.9)	
Palestine	330 (78.2)	92 (21.8)	
Qatar	234 (76.5)	72 (23.5)	
Iraq	196 (53.3)	172 (46.7)	
**I advise other parents to vaccinate their children against COVID-19**			**< 0.001**
No	1020 (83.6)	200 (16.4)	
Yes	706 (48.6)	747 (51.4)	
Neutral	824 (76.9)	247 (23.1)	
**COVID-19 vaccine is necessary for those younger than 18 years**			**< 0.001**
No	1318 (81.1)	308 (18.9)	
Yes	654 (49.8)	659 (50.2)	

٭Chi square test

### Parents’ attitudes towards COVID-19 vaccine according to children’s vaccination status

Table 5 presents parents’ attitudes towards COVID-19 vaccine according to children’s vaccination status. All attitudes’ items had significant statistical associations with vaccination status (*p* ≤ 0.05). About half of parents who believed that COVID-19 vaccines are safe for children had vaccinated their offspring compared to only about 20% of parents who did not have the same belief (*P* <  0.001). In addition, parents who were against children’s vaccines due to the unknown long-term side effects of the vaccine (*P* <  0.001) or perceived a lack of scientific research about the effects of the vaccine on children (*P* <  0.001) were less likely to vaccinate their children. Most importantly, willingness to vaccinate children decreased among parents who think that the vaccine may change human genes (*P* <  0.001).

**Table 5:** Parents’ attitudes towards COVID-19 vaccination of children according to children vaccination status (*n* = 3744)

Variable	Children vaccination status		Total (n)	***P*** value٭
	No (%)	Yes (%)		
**COVID-19 vaccine protects children from the infection**				**< 0.001**
No	1092 (81.0)	256 (19.0)	1348	
Yes	646 (51.7)	633 (48.3)	1249	
Neutral	812 (70.8)	335 (29.2)	1147	
**COVID-19 vaccines are safe to be used on children**				**< 0.001**
No	1115 (80.4)	271 (19.6)	1386	
Yes	468 (50.2)	465 (49.8)	933	
Neutral	967 (67.9)	458 (32.1)	1425	
**I encourage vaccination of children against COVID-19**				**< 0.001**
No	1225 (80.9)	290 (19.1)	1515	
Yes	647 (52.0)	598 (48.0)	1245	
Neutral	678 (68.9)	306 (31.1)	948	
**A child can be infected with COVID-19 even after vaccination**				**< 0.001**
No	122 (68.5)	56 (31.5)	178	
Yes	1957 (69.6)	856 (30.4)	2813	
Neutral	471 (62.5)	282 (37.5)	753	
**A child who had COVID-19 infection do not need the vaccine**				**< 0.001**
No	646 (58.4)	460 (41.6)	1106	
Yes	1017 (74.9)	341 (25.1)	1358	
Neutral	887 (69.3)	393 (30.7)	1280	
**Natural immunity of the infection is better than vaccination**				**< 0.001**
No	450 (56.2)	351 (43.8)	801	
Yes	1539 (76.0)	487 (24.0)	2026	
Neutral	561 (61.2)	356 (38.8)	917	
**A healthy child does not require COVID-19 vaccine**				**< 0.001**
No	672 (57.5)	496 (42.5)	1168	
Yes	1250 (76.0)	395 (24.0)	1645	
Neutral	628 (67.5)	303 (32.5)	931	
**COVID-19 is not serious and does not require a vaccine**				**< 0.001**
No	1115 (60.6)	725 (39.4)	1840	
Yes	606 (77.5)	176 (22.5)	782	
Neutral	829 (73.9)	293 (26.1)	1122	
**Face masks, social distancing, and sterilization are enough to prevent spread of COVID-19**				**< 0.001**
No	644 (61.9)	397 (38.1)	1041	
Yes	1384 (72.1)	535 (27.9)	1919	
Neutral	522 (66.6)	262 (33.4)	784	
**I’m against all children vaccines, not only the COVID-19 vaccine**				**< 0.001**
No	1582 (65.0)	852 (35.0)	2434	
Yes	466 (77.8)	133 (22.2)	599	
Neutral	502 (70.6)	209 (29.4)	711	
**COVID-19 vaccine should only be given to elderly and children with diseases**				**< 0.001**
No	943 (59.9)	631 (40.1)	1574	
Yes	841 (74.2)	293 (25.8)	1134	
Neutral	766 (73.9)	270 (26.1)	1036	
**The benefit of COVID-19 vaccine outweighs its side effects**				**< 0.001**
No	757 (81.0)	178 (19.0)	935	
Yes	805 (58.2)	577 (41.8)	1382	
Neutral	988 (69.2)	439 (30.8)	1427	
**I’m against children vaccine as long-term side effects of the vaccine aren’t known**				**< 0.001**
No	411 (52.6)	370 (47.4)	781	
Yes	1407 (78.0)	397 (22.0)	1804	
Neutral	732 (63.2)	427 (36.8)	1159	
**Children should be vaccinated against COVID-19 only when advised by a physician**				**< 0.001**
No	851 (81.4)	195 (18.6)	1046	
Yes	969 (58.2)	696 (41.8)	1665	
Neutral	730 (70.7)	303 (29.3)	1033	
**The government have the right to impose COVID-19 vaccine on all citizens**				**< 0.001**
No	1471 (78.3)	408 (21.7)	1879	
Yes	570 (55.4)	458 (44.6)	1028	
Neutral	509 (60.8)	328 (39.2)	837	
**COVID-19 vaccine will end the pandemic**				**< 0.001**
No	1193 (78.7)	322 (21.3)	1515	
Yes	481 (54.4)	404 (45.6)	885	
Neutral	876 (65.2)	468 (34.8)	1344	
**Children should not be vaccinated against COVID-19 because vaccination is painful**				**< 0.001**
No	1276 (62.2)	777 (37.8)	2053	
Yes	536 (77.8)	153 (22.2)	689	
Neutral	738 (73.7)	264 (26.3)	1002	
**Children should not be vaccinated against COVID-19 due to lack of scientific research about effects of the vaccine on children**				**< 0.001**
No	363 (52.2)	332 (47.8)	695	
Yes	1568 (77.8)	448 (22.2)	2016	
Neutral	619 (59.9)	414 (40.1)	1033	
**COVID-19 vaccine is not effective because of the frequent mutations of the virus**				**< 0.001**
No	525 (57.5)	388 (42.5)	913	
Yes	1131 (78.5)	309 (21.5)	1440	
Neutral	894 (64.3)	497 (35.7)	1391	
**COVID-19 vaccine changes human genes**				**< 0.001**
No	891 (63.0)	523 (37.0)	1414	
Yes	472 (77.8)	135 (22.2)	607	
Neutral	1187 (68.9)	536 (31.1)	1723	

٭Chi square test

### Regression analysis of factors associated with increased chance of having vaccinated children

In the binary regression analysis, the vaccination status was categorized into two main levels; (Yes) and (No). Children’s vaccination was significantly correlated with parents’ age, education, occupation, parents’ previous COVID-19 infection, and their vaccination status. Participants aged ≥50 years and those aged 40-50 years had an OR of 18 (OR = 17.9, CI: 11.16-28.97) and 13 (OR = 13.2, CI: 8.42-20.88) for vaccinating their children compared to those aged 18-29 years. Significantly, parents who had COVID-19 vaccine were five folds more likely to vaccinate their children compared with parents who didn’t receive the vaccine (OR = 4.9, CI: 3.12-7.70). Table 6 presents the results of the binary logistic regression analysis.

**Table 6:** Logistic regression analysis of factors associated with increased odds of having vaccinated children

Variable	Odds ratio	95% Confidence interval		***P*** value
		Lower	Upper	
**Participant’s age**				
18-29	Ref ^a^			
30-39	2.2	1.43	3.62	< 0.001
40-40	13.2	8.42	20.88	< 0.001
≥ 50	17.9	11.16	28.97	< 0.001
**Participant’s education**				
Secondary or less	2.1	1.46	2.79	< 0.001
≥ Graduate	Ref ^a^			
**Work or study field**				
None health-related	1.3	1.06	1.52	0.009
Health-related	Ref ^a^			
**Had COVID-19 before**				
No	1.3	1.07	1.47	0.005
Yes	Ref ^a^			
**Received a COVID-19 vaccine**				
No	Ref ^a^			
Yes	4.9	3.12	7.70	< 0.001
Intend to get the vaccine	2.7	1.49	4.94	0.001
Don’t intend to get the vaccine	1.8	1.01	3.35	0.044

^a^ Reference for other categories

## Discussion

### Prevalence of children vaccination against COVID-19 and parents’ beliefs about COVID-19 vaccine safety

Globally, there is a dearth of literature regarding the vaccine coverage amongst children and most published studies have assessed the parents’ willingness or intention to vaccinate children rather than true vaccination status. The preliminary finding of this study shows that most of the parents in the Arab countries are hesitant to vaccinate their children. This hesitancy can be explained by the lack of information about vaccine safety [25], concerns about vaccine effectiveness [26]. However, this finding might be different from other studies conducted in different countries. For example, results from a previous study in the Singapore population showed much higher willingness of parents to vaccinate their children aged between 12 and 18 years [26]. The variance between our study and the Singaporean study can be explained by differences in culture and society. Despite the majority of the parents have been already vaccinated against COVID-19, most of them didn’t vaccinate their children against COVID-19. This finding cannot be isolated from the other finding which demonstrated that one third of the participants believe that all vaccines are not safe.

### The relationship between parents’ socio-demographic characteristics and vaccination status of their children

Several significant correlations between children’s vaccination status and participants’ sociodemographic characteristics were found in this study. For instance, older parents showed extreme higher readiness to vaccinate their children against COVID-19 than younger counterparts. This result is congruent with a previous study which found that parents younger than 40 are more hesitant to vaccinate children [27]. However, this finding mismatches a previous study which showed that parents younger than 35 in Latin American countries are less likely to be hesitant to vaccinate children [28]. In terms of children’s number, parents with three or more children were more willing to vaccinate them than their counterparts. Participants of children aged between 12 and 17 years were more ready to vaccinate them than their counterparts. Our findings might be explained by the fact that younger parents usually have younger children (less than 12 years). Despite its approval, vaccination of the children younger than 12 years is still questionable among public.

In terms of caretaker’s gender, fathers showed a higher tendency towards vaccinating their kids than mothers. This result is consistent with a previous study from Lebanon [16] and US [29]. However, the result was different from a previous study in Poland which showed that mothers are more enthusiastic to vaccinate children than fathers [21]. This may be explained by the difference in the social role of fathers or mothers between communities.

Interestingly, parents whose work or study was health-related showed a lower tendency towards vaccinating their children than parents working in non-health-related fields. This finding could mean means that healthcare workers are concerned about safety, not convinced about efficacy, and worried about side effects. This result is consistent with a previous study stated that fewer caregivers plan to vaccinate their children against COVID-19 [30]. Also, parents with a lower educational level showed higher readiness towards vaccinating their children than parents with a higher educational level. This finding is consistent with a previous study which showed that undergraduate parents are more enthusiastic to vaccinate children than parents with higher education [12, 28, 31]. This finding is also congruent with previous studies which concluded that parents’ educational level is one of the determinants of willingness to vaccinate children [20, 32].

Parents who haven’t been infected with COVID-19 were more eager to vaccinate their children than other participants. This finding can be explained by their carefulness from being infected with COVID-19. Another explanation to this result may refer to the fear of the vaccine’s side effects perceived by those who have already got the infection before as the side effects can be similar to symptoms of the infection itself. This result is supported by previous studies. For example, a study showed that parents who are anxious from COVID-19 are keener to vaccinate children [31]. Another study showed that the perceived danger of being infected with COVID-19 is one of the determinants for parents’ willingness to vaccinate children [18]. Additionally, another research found that the readiness to get the COVID-19 vaccine increases with a higher perceived susceptibility to COVID-19 [33]. Vaccinated parents and those who intend to get the vaccine in the future were more willing to vaccinate their children than unvaccinated parents and those who do not intend to get the vaccine. The reason behind this difference can be explained by awareness of vaccinated parents about the COVID-19 vaccines which is supported by an earlier study [31].

Results of the current study showed that parents who had received COVID-19 vaccine or those who intend to get the vaccine are more likely to vaccinate their children. These results are consistent with results from another study stated that the likelihood of child vaccination was greater among parents who had already received or were likely to receive a COVID-19 vaccine [34].

### Parents’ attitudes towards vaccinating their children against COVID-19

This study showed that parents’ attitudes towards vaccinating children are significantly associated with their children’s vaccination status. For example, almost half of parents who believe that COVID-19 vaccines are safe on children had vaccinated their children. This result is similar to the rates found in a previous scoping review [32]. On the other hand, only one-fifth of parents who don’t trust safety of COVID-19 vaccines on children had vaccinated their children.

This study demonstrated that parents who were against children vaccination because of the unknown long-term side effects of the vaccine were less willing to vaccinate their children. This finding matches a previous research which found that one of the main parents’ concerns is the possible future complications of the vaccine [21]. Similarly, parents who think that the vaccine may change human genes were less eager to vaccinate their children. These findings were consistent with a previous study which showed that vaccination concerns determine parents’ willingness to vaccinate children against COVID-19 [26]. These results are consistent with results from Palestine which reported that adequate information about vaccines and their risk–benefit ratios are important to build trust and favorable attitudes towards vaccines [17].

It is worthy to mention that the willingness towards vaccinating children is significantly different depending on the country of residence. For instance, parents from KSA, UAE, Kuwait, and Iraq were more enthusiastic to vaccinate their children than those from Jordan, Lebanon, Palestine, and Qatar. A previous study has shown high rates of vaccine hesitancy in Jordan and Kuwait [35]. A probable cause of vaccination hesitancy may be also linked to misleading information shared on media platforms. This explanation was addressed in a previous study done on adolescents and parents of adolescents in the USA which showed that social media negatively impacted the opinions of adolescents and parents on COVID-19 vaccination [36].

### Strengths and limitations

The main strength of our study lies in the relatively large sample selected from eight middle eastern countries. This type of multi-country study gives importance to the results and overcomes the bias that might result from cultural, economic, or social varieties. However, the authors recognize that our cross-sectional design and convenient sample could impact statistical conclusions and the generalizability of the findings. Another limitation is the lack of precise data about the COVID-19 vaccines’ availability for children in the studied countries. Such data would address the role of vaccines’ availability in vaccinating children against the COVID-19. Although our results, collected via social media yielded a similar male-to-female ratio in the Arab world, the decisional autonomy in relation to children’s vaccination might refer finally to fathers. Additionally, there are many assumptions and findings in our study that can be fully understood, addressed, and explored with qualitative research which uses smaller information-rich samples and asks open questions to get a holistic picture about the factors that impact children vaccination against COVID-19. Moreover, our study reported the parents’ self-reported coverage of children vaccination against COVID-19, therefore, the method of ascertaining vaccination status of all children in the household could be affected by recall bias. Finally, other factors could impact COVID-19 vaccine uptake among children, including availability of vaccines, awareness of recommendations, access or convenience of the services and their booking systems. As these factors were not measured in our present study, it is complicated to infer what the largest barriers to uptake are.

## Recommendations

Face-to-face information or educational interventions should be used to help parents understand why vaccines are important; explain where, how, and when to access service and respond to hesitations and concerns about vaccine safety or effectiveness. Such interventions are interactive and can be tailored to target specific populations or identified barriers. Concise and clear vaccine information should be provided in multiple languages to improve vaccine confidence. Targeted campaigns should be implemented for parents that are socioeconomically disadvantaged and less educated.

## Conclusion

The parents’ self-reported coverage of children vaccination against COVID-19 in Arab countries seems to be much lower than those in other countries such as Singapore. Moreover, parents in the studied countries are still hesitant to vaccinate their children against the COVID-19. This hesitancy could be led by certain parents’ socio-demographic characteristics, contracting COVID-19 infection before, and their vaccination status. To increase COVID-19 vaccination rates in the countries located in the EMR, it is essential to address variables connected to parents’ hesitancy to vaccinate children.

## Supplementary Information

**Additional file 1: Supplementary File.**

## Data Availability

The data presented in this study are available on request from the corresponding author (MK).

## Abbreviations

COVID-19: Coronavirus Disease-2019
EMR: Eastern Mediterranean Region
KSA: Kingdom of Saudi Arabia
OR: Odd Ratio
PHEIC: Public Health Emergency of International Concern
UAE: United Arab Emirates
