# Supplementary material for: Children’s rates of COVID-19 vaccination as reported by parents, vaccine hesitancy, and determinants of COVID-19 vaccine uptake among children: a multi-country study from the Eastern Mediterranean Region
Source: BMC Public Health. 2022 Jul 18;22:1375. doi: 10.1186/s12889-022-13798-2 (PMC9294741; doi:10.1186/s12889-022-13798-2)
Supplement: Supplementary file 1 — Additional file 1: Supplementary File. [file 12889_2022_13798_MOESM1_ESM.pdf]

Supplementary File: Children's Rates of COVID-19 Vaccination as Reported by Parents, Vaccine Hesitancy, and Determinants of COVID-19 Vaccine Uptake Among Children: A Multi-Country Study from the Eastern Mediterranean Region

---

1. Gender:
   - Male
   - Female
2. Age:
   - 18-29
   - 30-39
   - 40-49
   - ≥50
3. Marital status:
   - Single
   - Married *Skip to question 2*
   - Other *Skip to question 2*
4. Education level:
   - High school or below
   - Graduate or post graduate
5. Work or study is
   - Health related
   - Non health related
6. Country you currently live in ……………………………………
7. Previous COVID19 infection:
   - Yes
   - No
8. Did you take the vaccine?
   - Yes *Skip to question 10*

- No

9. Do you intend to be vaccinated?

- Yes
- No
- Maybe

10. Do you have children aged ≤ 18 years?

- Yes
- No

11. How many children do you have?

- 1
- 2
- 3 or more

12. Age of your children?

- between 12-17 years
- less than 12 years
- I have children in both age groups

13. Did any of your children receive the vaccine?

- Yes *Skip to question 13*
- No *Skip to question*

14. Why did they take the vaccine?

Tick all that apply.

- ☐ Mandatory for school
- ☐ Mandatory for travelling
- ☐ Forced by governmental regulations
- ☐ To protect them/the family
- ☐ Influenced recommended by friends/relatives
- ☐ My child is in high-risk group
- ☐ Recommended by a physician
- ☐ To reduce transmission in schools

15. Do you think vaccine is needed for those aged ≤ 18 years?

- Yes
- No
- I don't know

16. What brand do you feel is safer for this age group?

Tick all that apply.

- [ ] Pfizer (American)
- [ ] Sinopharm
- [ ] Astrazeneca Moderna
- [ ] Sputnik
- [ ] All are safeNone

17. Does having the choice of vaccine type will affect your decision

- Yes
- No
- Neutral

18. Would you advise other parents to vaccinate their children?

- Yes
- No
- Neutral

19. Attitude towardsCOVID19 vaccination

The following questions ask about attitudes toward COVID-19 vaccination for children younger than18 years. You will find statements in the list. ***Please mark only one choice as follows:***

1. **Disagree**

2. **Neutral**

3. **Agree**

	1	2	3
1. COVID-19 vaccines protect children from COVID-19 infection	⬭	⬭	⬭
2. COVID-19 vaccines are safe for children.	⬭	⬭	⬭
3. I encourage children's vaccination against COVID-19.	⬭	⬭	⬭
4. COVID-19 infection can occur even after vaccination.	⬭	⬭	⬭
5. Children who were infected with COVID-19 do not need to get vaccinated	⬭	⬭	⬭
6. Natural immunity is better than the vaccine	⬭	⬭	⬭
7. Healthy children do not need a vaccine against COVID--19.	⬭	⬭	⬭
8. COVID--19 is exaggerated, it's not a risky disease, so no vaccination is needed	⬭	⬭	⬭

9. Masks, hygiene, social distance, and other protective measures are enough

10. I oppose all vaccines for children. ((Not only COVID-19 vaccine)

11. Vaccination is only needed for the elderlyand those with chronic illnesses ((like children with asthma))

12. COVID-19 vaccine benefits outweigh the side effects.

13. Children should not be vaccinated because the long--term effects off vaccinationare unknown.

14. Children must be vaccinated if it was recommended by a physician/MOH

15. The government has the right to forceeveryone to get COVID--19 vaccine.

16. COVID--19 vaccine will end the pandemic.

17. Children should not be vaccinated because vaccination is painful.

18. Children should not be vaccinated due to lack off scientific studies about COVID--19 vaccination on children.

19. Vaccine is Not effective due to frequent mutations
